# Calorimetric Studies of the Mg-Pt System

**DOI:** 10.3390/ma18133075

**Published:** 2025-06-28

**Authors:** Adam Dębski, Magda Pęska, Sylwia Terlicka, Julita Dworecka-Wójcik, Władysław Gąsior, Wojciech Gierlotka, Andrzej Budziak, Marek Polański

**Affiliations:** 1 Institute of Metallurgy and Materials Science, Polish Academy of Sciences, 25, Reymonta Street, 30-059 Kraków, Poland; 2 Department of Functional Materials and Hydrogen Technology, Military University of Technology, 2 Kaliskiego St., 00-908 Warsaw, Poland; 3 Department of Materials Science and Engineering, National Dong Hwa University, Hualien 974301, Taiwan; 4 Faculty of Energy and Fuels, AGH University of Krakow, Al. Mickiewicza 30, 30-059 Kraków, Poland

**Keywords:** intermetallics, enthalpy, thermodynamic properties, calorimetry, X-ray diffraction, Mg–Pt

## Abstract

This study presents the limiting partial enthalpy of a solution of Pt in liquid Sn and Al baths, as well as, for the first time, the standard enthalpies of the formation of intermetallic phases and alloys of the Mg–Pt system, obtained using solution calorimetry. The alloys were prepared via mechanical alloying and subsequently examined via X-ray diffraction (XRD) and scanning electron microscopy (SEM). The limiting partial enthalpy of a solution of Pt in liquid baths was measured at 931 K and 1033 K in the Sn bath and at 1036 K in the Al bath. The measured values are negative and equal to −126.0 ± 3.4 kJ/mol and −126.3 ± 3.5 kJ/mol at 931 K and 1033 K, respectively, in the Sn bath and −217.9 ± 1.2 kJ/mol in the Al bath. Subsequently, the measured heat effects were used to calculate the standard enthalpies of the formation of the intermetallic phases. The resulting values are as follows: −29.5 ± 1.8 kJ/mol·at. for Mg_6_Pt, −53.9 ± 1.6 kJ/mol·at. for Mg_3_Pt, −65.2 ± 0.4 kJ/mol·at. for Mg_2_Pt, −78.7 ± 2.1 kJ/mol·at. for MgPt, −44.5 ± 0.4 kJ/mol·at. for MgPt_3_, and −26.4 ± 1.0 kJ/mol·at. for MgPt_7_. These values of the standard enthalpies of the formation of the intermetallic phases were compared with available ab initio data and those calculated using Miedema’s model. The data obtained using Miedema’s model were the least exothermic compared to the data obtained from calorimetric measurements and other theoretical calculations.

## 1. Introduction

Electricity is a fundamental source of energy in modern/contemporary society and the economy. Both professional and personal aspects of life, as well as economic prosperity, rely heavily on its uninterrupted supply. Consequently, the electricity demand continues to grow steadily year by year. Conventional energy sources such as oil and coal are finite and are expected to be depleted soon. Therefore, a substantial increase in the share of energy from renewable sources is imperative to achieve the so-called “green transition.” This transition has the potential to significantly mitigate the adverse effects of global climate change, air pollution, and the growing risks of power supply interruptions and price volatility. However, this requires decisive and rapid actions to promote and develop greenhouse gas emission-free energy technologies.

Hydrogen is considered one of the most promising clean energy carriers. To play a key role in the energy transition, however, it must be widely adopted in sectors in which it is currently almost absent, such as transportation, construction, and power generation. One of the most critical challenges of hydrogen technology is the safe, efficient, and economical storage of large quantities of hydrogen. In this context, hydrogen storage in the form of hydrides in magnesium-based alloys, which have been extensively studied since the 1950s, remains one of the main technologies for high-temperature hydrogen storage. Numerous scientific studies confirm that the addition of small amounts of noble metals to magnesium-based alloys can significantly improve the kinetics of hydrogen sorption/desorption and the thermodynamic stability of hydrides and reduce operating temperatures [1,2]. Although the use of large quantities of these metals as additives to solid hydrogen storage materials is economically unviable, the interaction of Mg–Pt alloys with hydrogen remains important due to potential applications in other industrial areas, such as catalysis.

The design and development of new functional materials require a thorough understanding of their thermodynamic properties. However, comprehensive data on the thermodynamic and physicochemical properties of the Mg–Pt system are still scarce in the literature. In their review article, Nayeb-Hashemi and Clark [3] compiled information on the crystal structure and lattice parameters of five known intermetallic phases at the time, namely, Mg_6_Pt, Mg_3_Pt, MgPt, MgPt_3_, and MgPt_7_, as reported in earlier works [4,5,6]. Later, Range and Hafner [7] redefined the structural parameters for the MgPt_3_ phase. Furthermore, Schmitt et al. [8] described the crystal structure of a newly discovered intermetallic compound Mg_2_Pt, which is synthesized by reacting pure elements in a sealed niobium container.

Preliminary thermodynamic data for the Mg–Pt system, including solid phases and alloys, were presented by Wengert and Spanoudis [9], who estimated the upper limit of the Gibbs free energy of formation for MgPt_7_ and MgPt_3_ by evaluating the feasibility of reactions leading to their formation. Standard Gibbs free energies of formation for these two intermetallic compounds were determined by Jacobs et al. [10] using solid-state galvanic cell measurements in the temperature range of 950–1200 K.

**Figure 1:**
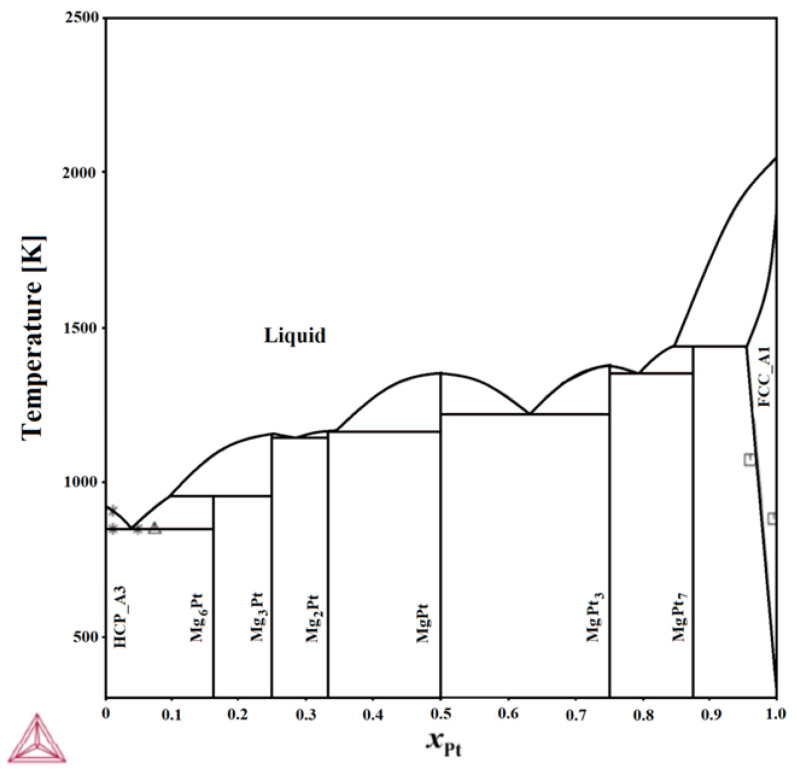
Calculated phase diagram of the Mg–Pt system [13]. 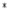 [3]; 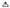 [4]; 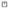 [5].

Our group has made some efforts to fill the existing data gaps in the Mg–Pt system. In a previous study [11], the enthalpy of mixing liquid Mg–Pt alloys was reported for the first time in a concentration range up to 0.19 at.% Pt at a temperature of 1031 K. Negative mixing enthalpies were observed throughout the entire investigated range. Additionally, ab initio calculations [12] have been published, predicting the thermodynamic properties of all intermetallic phases known in the Mg–Pt equilibrium system. In our most recent work on this system, a preliminary phase diagram was proposed for the first time [13] (Figure 1).

Solution calorimetry is a well-known and extensively used technique for the experimental determination of thermodynamic properties, including the enthalpy of the formation of intermetallic compounds. When performed on samples with phase purity verified via XRD analysis, it offers a high degree of accuracy and reliability.

In the present article, the standard enthalpy of formation of Mg–Pt intermetallic phases and alloys, determined calorimetrically for samples synthesized by mechanical alloying, is reported for the first time. Furthermore, the partial limiting enthalpy of the dissolution of Pt in liquid Sn and Al was also measured at 931 and 1033 K (for liquid Sn baths) and 1033 K (for a liquid Al bath).

## 2. Materials and Methods

**Figure 2:**
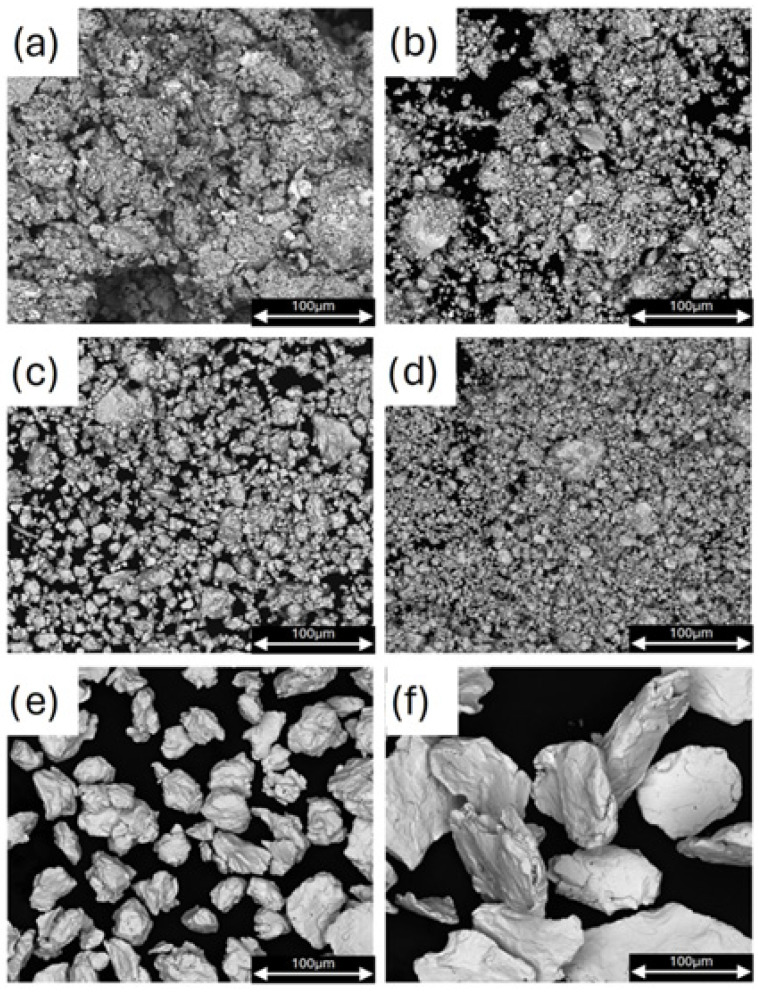
Morphological characteristics of Mg–Pt powder particles after the mechanical synthesis and annealing of the alloys: (**a**) S1 (Mg6Pt) (**b**) S2 (Mg3Pt) (**c**) S3 (Mg2Pt) (**d**) S4 (MgPt) (**e**) S5 (MgPt3), (**f**) S6 (MgPt7).

Table 1 provides details on the purity and suppliers of the materials used for sample preparation in the Mg–Pt system. To produce six samples of the Mg–Pt system (S1 (Mg_6_Pt), S2 (Mg_3_Pt), S3 (Mg_2_Pt), S4 (MgPt), S5 (MgPt_3_), and S6 (MgPt_7_)), the mechanical alloying (MA) method was used. For this purpose, appropriate amounts of magnesium powder and cut platinum wire (0.1 mm in diameter; Safina Pol., 99.99%) were weighed. Mechanical alloying (MA) was performed using a planetary ball mill (Fritsch Pulverisette 7, Idar-Oberstein, Germany) operating at a rotational speed of 800 rpm for 1 h. A 20 mL stainless steel vial and ten AISI 304 stainless steel balls (Ø10 mm) were used as the milling medium. The preparation of the vial with the powders was carried out in a glove box under an inert gas atmosphere (<1 ppm O_2_ and H_2_O). Following the ball milling procedure, the milled powder was enclosed in a specially designed stainless steel reactor sealed with a copper gasket to ensure a tight closure. To reduce the risk of powder oxidation, the annealing furnace was operated inside a LabMaster (MBraun, Munich, Germany) glovebox. A similar procedure had been successfully applied previously to samples from the Mg–Pd system [14]. The prepared samples were then annealed at 500 °C for one hour and subsequently cooled slowly together with the furnace. Following the annealing process, the resulting Mg–Pt alloys were examined using X-ray diffraction (XRD). For this purpose, a Rigaku Ultima IV (Tokyo, Japan) powder diffractometer equipped with a cobalt anode X-ray tube (λ = 1.78 Å) was used. Measurements were conducted at 40 mA and 40 kV, with a scanning rate not exceeding 1°/min. The instrument utilized cross-beam optics (CBO), parallel beam geometry, and a fast linear detector (Detex Ultra, Rigaku, Tokyo, Japan), resulting in diffraction patterns with very low Kβ contributions. The obtained diffraction data were analyzed using the PDXL2 2.8.4.0 s software package. Subsequently, the morphology of the powders obtained after mechanical alloying was examined using high-resolution field emission scanning electron microscopy (FE-SEM, FEI Quanta 3D, Hillsboro, OR, USA). Figure 2 presents the morphology of powder particles after mechanical synthesis and annealing. Observations of backscattered electrons (BSE) reveal particle features typical of samples milled in ball mills.

The standard enthalpies of formation (Δ_f_*H*) of the obtained alloys were measured using the MHTC 96 Line Evo drop calorimeter produced by Setaram (Setaram Instrumentation—KEP technologies, Caluire, France). The observed heat effects were registered using Calisto software (1.39). All experiments were carried out using alumina crucibles, and the measurement procedure followed the methodology established in our previous calorimetric studies [14]. The procedure is as follows: first, a weighed amount of Sn or Al bath (approximately 30 g and 15 g, respectively) was placed into the experimental alumina crucible and inserted into the calorimeter before starting a series of measurements. Then, the calorimeter was assembled, evacuated multiple times by a vacuum pump, and flushed with high-purity Ar. After these initial steps, the instrument was heated to the measurement temperature, and the calibration constant was determined using approximately six pieces of high-purity tin or aluminum (depending on the used calorimetric bath; the mass of such samples was about 200 mg for Sn and 50 mg for Al). After obtaining the calibration constant values, the small pieces of the samples prepared by mechanical alloying were consecutively added into the bath. The heat effect of the dropped samples was recorded until the baseline value was the same as before the dropping. All measurements were performed in a high-purity argon atmosphere (Table 1).

The standard enthalpy of formation (Δ_f_*H*) of the tested alloys at 298 K was determined by calculating the difference in thermal effects associated with heating the samples from room temperature (298 K) to the measurement temperature, followed by their dissolution in the metallic bath. The Δ_f_*H* value was obtained using the following equation:

(1) $${∆}_{f}H=x_{Mg}∆H_{Mg}^{0}+x_{Pt}∆H_{Pt}^{0}-∆H_{x_{Mg}x_{Pt}}^{0}$$

where Δ_f_*H* is the standard enthalpy of formation of the studied alloys; *x_Mg_* and *x_Pt_* are the mole fractions of the components Mg and Pt, respectively; and ${∆H}_{Mg}^{0}$, $∆H_{Pt}^{0}$, and $∆H_{x_{Mg}x_{Pt}}^{0}$ are the heat effects that accompany the dissolution of one mole of each of the components (Mg and Pt) and alloy in the liquid bath, respectively. The $∆H_{Mg}^{0}$ and $∆H_{Pt}^{0}$ values are the sums of the limiting partial enthalpy of the solution of liquid Mg (${∆}_{sol}{\bar{H}}_{Mg(l)\;}^{\infty }$) and Pt (${∆}_{sol}{\bar{H}}_{Pt(l)\;}^{\infty }$) in a liquid Sn or Al bath and the enthalpy change of the pure Mg and Pt from room temperature to the measurement temperature. ${\Delta H}_{Mg}^{T_{D}\to T_{M}}$ and ${\Delta H}_{Pt}^{T_{D}\to T_{M}}$ are the differences in the molar enthalpy of Mg or Pt between the room (*T_D_*) and measurement (*T_M_*) temperatures calculated using relations [15].

(2) $${∆H_{Mg}^{0}=∆}_{sol}{\bar{H}}_{Mg(l)\;}^{\infty }+\Delta H_{Mg}^{T_{D}\to T_{M}}$$

(3) $${∆H_{Pt}^{0}=∆}_{sol}{\bar{H}}_{Pt(l)\;}^{\infty }+\Delta H_{Pt}^{T_{D}\to T_{M}}$$

In this study, the heat effects of the dissolution of the Mg–Pt alloys ($∆H_{x_{Mg}x_{Pt}}^{0}$), as well as the heat effects of the dissolution of platinum in liquid Sn or Al bath, were measured.

## 3. Results and Discussion

### 3.1. Phase Analysis and Microstructural Characterization

**Figure 3:**
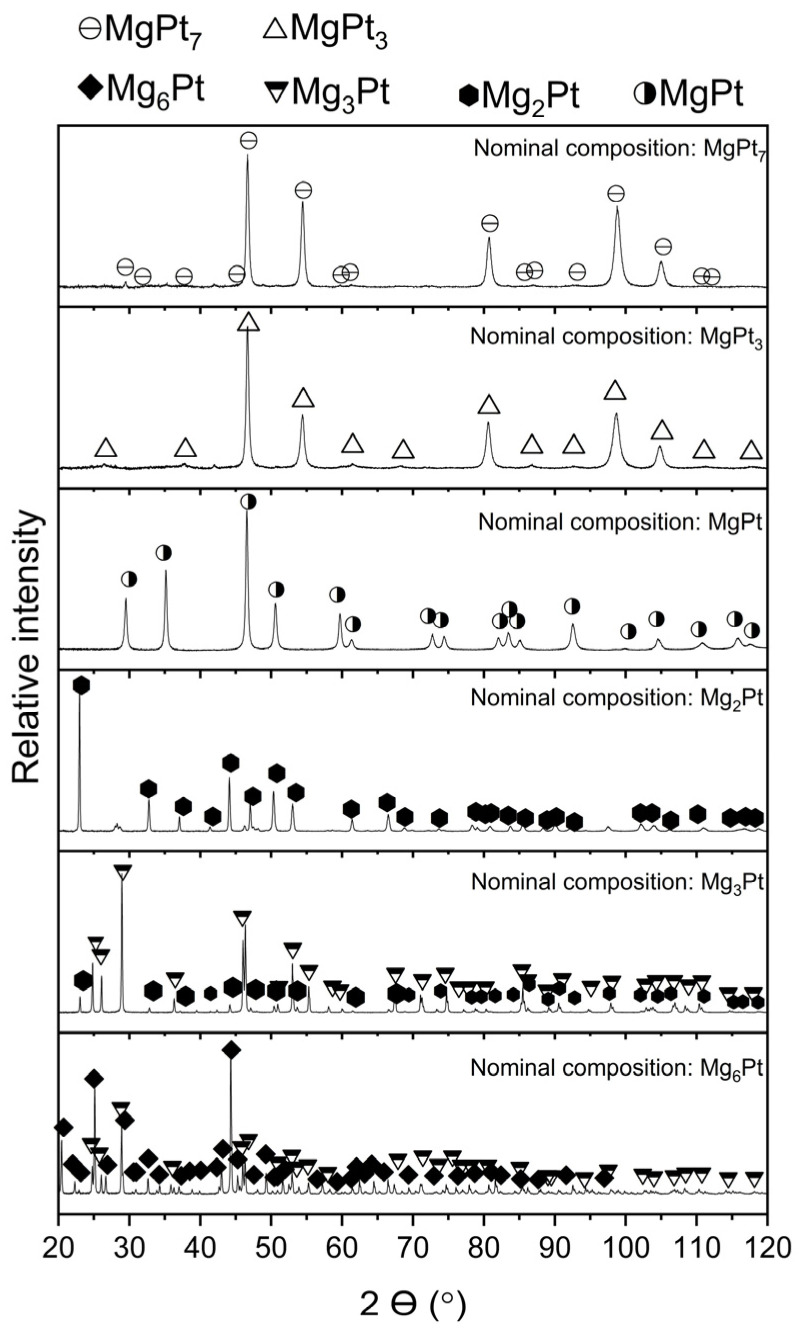
Phase composition of Mg–Pt samples based on XRD analysis after mechanical alloying and subsequent annealing.

X-ray diffraction (XRD) was employed to examine the synthesized Mg–Pt alloys, with the resulting diffractograms shown in Figure 3. For the alloy with a nominal composition corresponding to the Mg_6_Pt phase, characteristic diffraction peaks of this phase were identified. However, additional reflections corresponding to the Mg_3_Pt phase were also observed. The X-ray diffraction pattern of the alloy with a nominal composition corresponding to the Mg_3_Pt phase showed intense reflections from the expected Mg_3_Pt phase, as well as reflections attributable to the Mg_2_Pt phase. The presence of the Mg_2_Pt phase was further confirmed in the diffraction pattern of the next alloy, designed to correspond to this specific phase. Nevertheless, several very low-intensity reflections were observed in the pattern, which could not be identified. It should be noted that microanalysis of the chemical composition did not reveal the presence of any other elements, excluding contamination. The alloy with a nominal composition corresponding to the MgPt phase exhibited reflections originating solely from the intended phase. It is worth mentioning that the phases identified in the X-ray diffraction patterns were referenced from the ICDD PDF-4+ database, except for the Mg_6_Pt phase. This phase was modeled by the authors based on its structural similarity to the Mg_6_Pd phase, assuming the same type of crystal lattice and positions of atoms. Based on this model, the identification of the synthesized Mg_6_Pt phase was carried out. A high degree of agreement was obtained between the simulated and experimental spectra, confirming the assumed structural similarity between the Mg_6_Pd and Mg_6_Pt phases.

### 3.2. Calorimetric Studies

**Figure 4:**
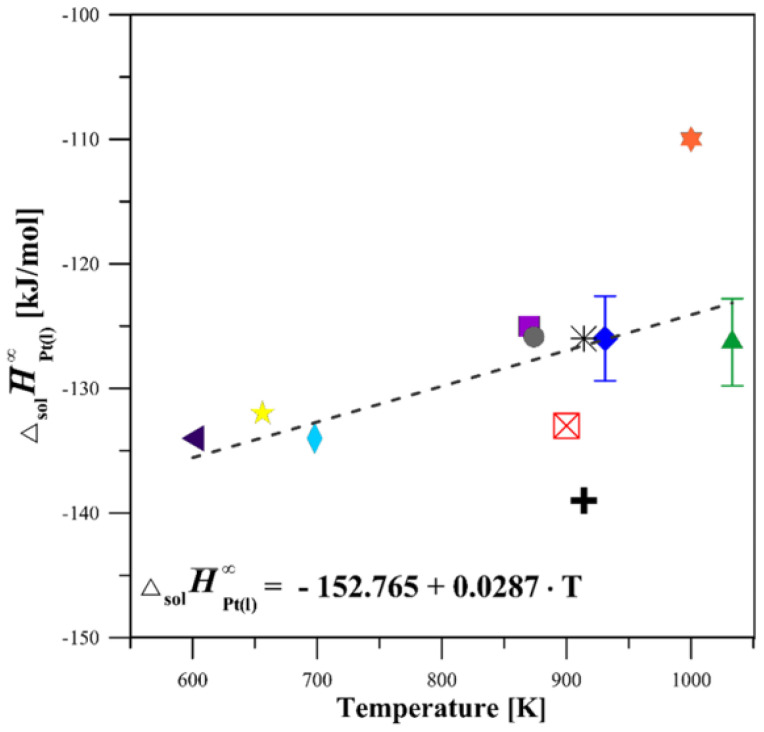
Limiting partial enthalpy of solution of Pt in liquid Sn: present results versus literature values [17,18,19,20,21,22,23,24] cited in [25]. 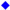 This study at 931 K; 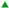 This study at 1033 K; 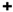 [17] at 914 K; 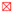 [18] at 900 K; 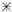 [19] at 914 K; 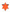 [2] at 1000 K; 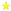 [21] at 656 K; 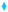 [22] at 698 K; 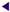 [23] at 600 K; 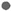 [24] at 874 K; 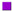 [25] at 870 K; 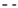 Average ${∆}_{sol}{\bar{H}}_{Pt(l)\;}^{\infty }$.

The limiting partial enthalpy of the solution of Pt (${∆}_{sol}{\bar{H}}_{Pt(l)\;}^{\infty }$) was determined at two temperatures (931 and 1033 K) in liquid Sn and at one temperature (1033 K) in liquid Al. Carrying out the experiments at these temperatures allowed for the complete dissolution of the samples in the solvent and provided safe and stable operating conditions for the calorimeter. The value of limiting partial enthalpies of solution of magnesium in a liquid tin was taken from our previous studies [16] and is equal to −8.6 ± 1.1 kJ/mol. The other essential data on the used metals were calculated with the use of Pandat 2013 software [15]. The obtained results for the limiting partial enthalpy of a solution of Pt in liquid Sn and Al were presented in Table 2 and Table 3, respectively. Moreover, the limiting partial molar enthalpies of Pt in the Sn bath are shown in Figure 4, together with the other data available in the literature [17,18,19,20,21,22,23,24,25].

Based on the results of this study, the limiting partial enthalpy of a solution of Pt in liquid Sn is found to be negative, amounting to −126.0 ± 3.4 kJ/mol at 931 K and −126.3 ± 3.5 kJ/mol at 1033 K (refer to Table 2 and Figure 4). The measured values of the ${∆}_{sol}{\bar{H}}_{Pt(l)\;}^{\infty }$ are in satisfactory agreement with the data presented in [19,24,25] and are slightly less exothermic than those appearing in works [18,21,22,23]. The lower exothermic values for ${∆}_{sol}{\bar{H}}_{Pt(l)\;}^{\infty }$ reported by [20], which are inconsistent with those obtained in this study and other literature data, are probably the result of the incomplete dissolution of the solute, hence such a large difference. The average results of the limiting partial enthalpy of a solution of Pt in liquid Al are characterized by a large negative value and are equal to −217.9 ± 1.2 kJ/mol at 1033 K and the infinite dilution. To the best of our knowledge, the limiting partial enthalpy of the Pt solution in liquid Al at this temperature has not been measured until now. Therefore, a comparison with the existing literature is not possible. The heat effects and the values of standard enthalpies of formation of the investigated alloys determined from them are shown in Table 4.

**Figure 5:**
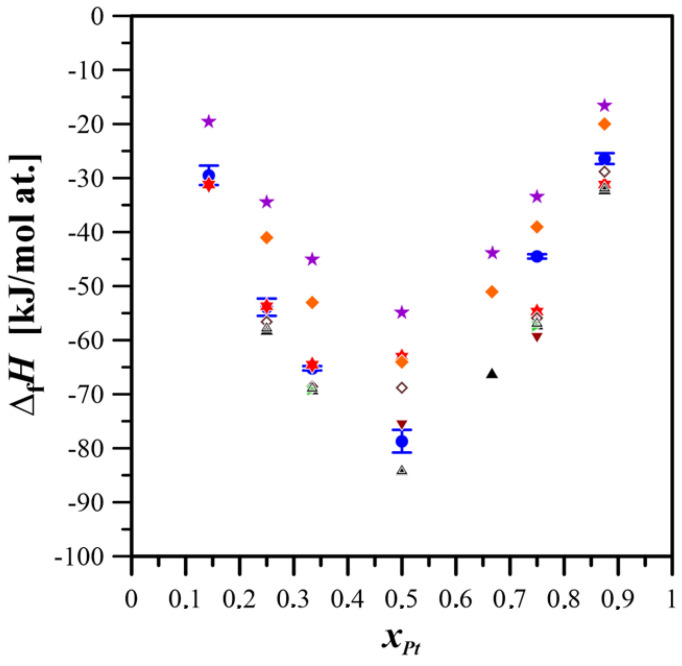
Comparison of the standard formation enthalpies for intermetallic phases and alloys of the Mg–Pt system obtained experimentally and through calculations, with results from ab initio methods and the Miedema model [26,27]. 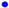 This study; 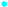 [10]; 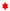 [13]; 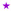 [26]; 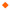 [28]; 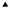 [29]; 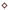 [30]; 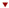 [31]; 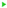 [32]; 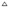 [33].

Figure 5 compares the formation enthalpies of the studied alloys, as determined in this work, with values reported in the literature.

The standard formation enthalpies of intermetallic compounds in the Mg–Pt system reported in this study exhibit closer agreement with ab initio calculations than with values predicted by the Miedema model. These results confirm the effectiveness of MA as a method for the synthesis of Mg–Pt alloys. The advantages of this technique become particularly apparent when we consider the limitations of conventional processing methods, such as melting and casting, which are hampered by the marked difference in the melting temperatures of magnesium and platinum, as well as the high vapor pressure of magnesium at the preparation temperature. These factors make it difficult to maintain the correct concentration of the constituents due to the rapid evaporation of magnesium and possible gravitational segregation during solidification caused by the significant density difference between magnesium and platinum. Mechanical alloying eliminates the problems mentioned above by enabling the creation of an alloy of a given composition entirely in the solid state.

## 4. Conclusions

The experimental results are presented for the limiting partial enthalpies of solutions of Pt in liquid Sn at 931 and 1033 K and in liquid Al at 1033 K. Furthermore, this work reports the first determination of the standard formation enthalpy of intermetallic phases in the Mg–Pt system through solution calorimetry. To prepare these intermetallic phases, the method of mechanical alloying was used. Owing to the high vapor pressure of magnesium, this method facilitates the synthesis of compounds that are otherwise extremely challenging or unfeasible to produce using conventional casting methods. Before the calorimetric measurements were conducted, structural analyses of the prepared samples were carried out.

The measured values of the limiting partial enthalpies of solutions of Pt in liquid Sn are generally consistent with available literature data. The experimentally determined standard enthalpy of the formation of the Mg–Pt intermetallic phases shows better agreement with ab initio calculation results than with those predicted by the Miedema model.

The obtained experimental data on the standard enthalpy of the formation of intermetallic phases in the Mg–Pt system not only contribute to a better understanding of the thermodynamic properties of this binary system but may also support future work involving theoretical modeling and application-oriented studies, especially in the context of hydrogen storage materials and the design of multi-component alloys.

## Figures and Tables

**Table 1:** Details of the materials employed.

Chemical Name	Source	Purity [Mass %]
Mg powder	Sigma Aldrich	>99
Pt wire	Safina a.s.	99.99
Ar	Pioniergas	99.9999

**Table 2:** Values for the limiting partial enthalpy of a solution of liquid Pt ${∆}_{sol}{\bar{H}}_{Pt(l)}^{\infty }$ in liquid Sn.

Measurement No.	Dropped Mass of Samples [g]	Amount of Pt in Sn Bath [at. %]	Heat Effect Δ*H*^ef^ [kJ/mol]	$\mathrm{Limiting}\;\mathrm{Partial}\;\mathrm{Enthalpy}\;\mathrm{of}\;\mathrm{Solution}\;{∆}_{sol}{\bar{H}}_{Pt(l)\;}^{\infty }$ [kJ/mol]
Series 1: atmosphere—Ar; calibration constant: *K* = 0.000003524 kJ/μVs; enthalpy of pure Pt: $\Delta H_{Pt}^{T_{D}\to T_{M}}\;$= 37.7333 kJ/mol; temperature of Sn bath: *T_M_* = 931 K; drop temperature: *T_D_* = 298 K; mass of Sn bath: *m*_Sn_ = 30.5011 g.				
1	0.0820	0.1633	−92.9	−130.7
2	0.0820	0.3261	−86.1	−123.8
3	0.0770	0.4785	−90.5	−128.2
4	0.0780	0.6324	−88.9	−126.6
5	0.0800	0.7897	−87.8	−125.5
6	0.0974	0.9806	−83.4	−121.1
Average	-	-	−88.2	−126.0
Standard error	-	-	3.4	3.4
Series 2: atmosphere—Ar; calibration constant: *K* = 0.000003377 kJ/μVs; enthalpy of pure Pt: $\Delta H_{Pt}^{T_{D}\to T_{M}}\;$= 40.8834 kJ/mol; temperature of Sn bath: *T_M_* = 1033 K; drop temperature: *T_D_* = 298 K; mass of Sn bath: *m*_Sn_ = 30.4691 g.				
1	0.0892	0.1778	−89.5	−130.4
2	0.0905	0.3576	−81.4	−122.3
3	0.0896	0.5350	−84.5	−125.4
4	0.0962	0.7247	−83.1	−124.0
5	0.0919	0.9052	−88.6	−129.5
6	0.0974	1.0959	−85.4	−126.2
Average	-	-	−85.4	−126.3
Standard error	-	-	3.5	3.5

**Table 3:** Values of the limiting partial enthalpy of a solution of liquid Pt ${∆}_{sol}{\bar{H}}_{Pt(l)}^{\infty }$ in liquid Al.

Measurement No.	Dropped Mass of Sample [g]	Amount of Pt in Sn Bath [at.%]	Heat Effect Δ*H*^ef^ [kJ/mol]	$\mathrm{Limiting}\;\mathrm{Partial}\;\mathrm{Enthalpy}\;\mathrm{of}\;\mathrm{Solution}\;{∆}_{sol}{\bar{H}}_{Pt(l)}^{\infty }$ [kJ/mol]
Atmosphere—Ar; calibration constant: *K* = 0.000003566 kJ/μVs; enthalpy of pure Pt: $\Delta H_{Pt}^{T_{D}\to T_{M}}\;$= 40.8834 kJ/mol; temperature of Al bath: *T_M_* = 1033 K; drop temperature: *T_D_* = 298 K; mass of Sn bath: *m*_Sn_ = 15.1001 g.				
1	0.1027	0.0940	−175.9	−216.8
2	0.0992	0.1846	−177.1	−218.0
3	0.1094	0.2843	−178.0	−218.9
Average	-		−177.0	−217.9
Standard error	-		1.2	1.2

**Table 4:** Heat effects, Δ*H*^ef^, and formation enthalpies, Δ_f_*H*, of the investigated alloys from the Mg–Pt system. Dissolution in liquid tin. Temperature of the Sn bath: 1036 K.

Sample	T [K]	Sample No.	Δ*H*^ef^ [kJ/mol at.]	Δ_f_*H* [kJ/mol at.]
S1 (Mg_6_Pt)	298	1	16.1	−30.7
		2	13.6	−28.2
		Average	14.9	−29.5
		Standard error	1.8	1.8
S2 (Mg_3_Pt)	298	1	31.6	−55.0
		2	29.3	−52.8
		Average	30.5	−53.9
		Standard error	1.6	1.6
S3 (Mg_2_Pt)	298	1	34.5	−64.9
		2	35.1	−65.4
		Average	34.8	−65.2
		Standard error	0.4	0.4
S4 (MgPt)	298	1	36.7	−80.9
		2	35.2	−79.3
		3	36.1	−80.3
		4	32.2	−76.3
		5	32.5	−76.6
		Average	34.5	−78.7
		Standard error	2.1	2.1
S5 (MgPt_3_)	298	1	−20.1	−44.8
		2	−20.6	−44.3
		Average	−20.3	−44.5
		Standard error	0.4	0.4
S6 (MgPt_7_)	298	1	−49.5	−25.7
		2	−48.1	−27.1
		Average	−48.8	−26.4
		Standard error	1.0	1.0

## Data Availability

The original contributions presented in this study are included in the article. Further inquiries can be directed to the corresponding authors.
